# Genes of the “regulation of lymphocyte activation” pathway may influence immune cells infiltration in growth hormone secreting pituitary tumors

**DOI:** 10.1007/s11102-025-01537-w

**Published:** 2025-05-26

**Authors:** Sabrina Chiloiro, Flavia Costanza, Giovanni Luca Scaglione, Filippo Russo, Carmela Nardelli, Antonella Giampietro, Pier Paolo Mattogno, Liverana Lauretti, Guido Rindi, Laura De Marinis, Marco Gessi, Antonio Bianchi, Francesco Doglietto, Ettore Domenico Capoluongo, Alfredo Pontecorvi

**Affiliations:** 1 Department of Medical and Surgical Translational Sciences, Catholic University of the Sacred Heart, 00168 Rome, Italy; 2 Pituitary Unit, Department of Endocrinology, Diabetology and Internal Medicine, Fondazione Policlinico Universitario Agostino Gemelli, Istituto di Ricovero e Cura a Carattere Scientifico (IRCCS), 00168 Rome, Italy; 3 Bioinformatic Unit, Istituto Dermopatico dell’Immacolata (IDI), Istituto di Ricovero e Cura a Carattere Scientifico (IRCCS), 00167 Rome, Italy; 4 Department of Molecular Medicine and Medical Biotechnologies, University of Naples Federico II, 80131 Naples, Italy; 5 CEINGE Biotecnologie Avanzate-Franco Salvatore S.C.A.R.L, 80145 Naples, Italy; 6 Department of Neurosurgery, Fondazione Policlinico Universitario A. Gemelli, Istituto di Ricovero e Cura a Carattere Scientifico (IRCCS), 00168 Rome, Italy; 7 Department of Woman and Child Health Sciences and Public Health, Anatomic Pathology Unit, Fondazione Policlinico Universitario A. Gemelli, Istituto di Ricovero e Cura a Carattere Scientifico (IRCCS), 00168 Rome, Italy; 8 Department of Clinical Pathology, San Giovanni-Addolorata Hospital, 00184 Rome, Italy

**Keywords:** Acromegaly, Pituitary adenomas, Genetics, Clinical exome sequencing, Tumor microenvironment, Immune cells infiltration

## Abstract

**Purpose:**

The tumor microenvironment (TME) may provide a useful framework for understanding the heterogeneous behavior of growth hormone (GH) secreting pituitary adenomas. Although the interest in TME in somatotropinomas has increased exponentially over the last few decades, there is limited elucidation of its mechanisms, particularly in relation to genes expression involved in its regulation.

**Methods:**

A retrospective, observational, single-center study was conducted on 85 subjects: 46 patients diagnosed with acromegaly and 39 controls. After DNA extraction, clinical exome sequencing was performed and genomic alterations were detected, classified, and filtered using a dedicated bioinformatics pipeline.

**Results:**

5759 unique genetic variants were found in patients with acromegaly. 33 patients (72%) showed the presence of at least one pathogenic variant in at least one of the following genes: FANCD2, SPTA1, TYRO3, and ZNF335. The enrichment pathway analysis of mutated genes was performed and showed that these genes were included in the same genetic pathway called “regulation of lymphocyte activation” (GO:0051249). Inflammatory infiltrate was analyzed in histological samples in 26 patients. A significantly higher number of CD68 + macrophages (P-value = 0.008), a lower number of CD8 + T lymphocytes (P-value = 0.037) and a higher CD68 + macrophages/ CD8 + T-lymphocytes ratio (P-value = 0.004) were observed in patients with pathogenic variants of genes of “regulation of lymphocyte activation” pathway.

**Conclusion:**

This study provides new insights into the genetic basis of the TME in somatotropinomas and suggests that genetics may influence immune cells infiltration in acromegaly.

**Supplementary Information:**

The online version contains supplementary material available at 10.1007/s11102-025-01537-w.

## Introduction

Acromegaly is a rare disease mostly caused by somatotropinomas, namely growth hormone (GH) secreting pituitary adenomas, due to persistently elevated serum levels of GH and, in turn, insulin-like growth factor I (IGF-I) [1–3]. Somatotropinomas are heterogeneous neoplasms for clinical behavior and outcomes [4, 5]. The pathogenesis of somatotropinomas remains uncertain, possibly due to the influence of various factors, including genetics, epigenetics, and the tumor microenvironment (TME) [6–9]. The TME may provide a useful framework for understanding the heterogeneous behavior of somatotropinomas, through the dynamic interplay between tumour cells and TME components [6–12]. This complex network has been shown to regulate the interaction between tumour cells and the host’s immune system, with the potential to modulate tumor behavior, including oncogenic mechanisms, tumor aggressiveness, and treatment response in acromegaly [6, 10, 11, 13]. The TME of somatotropinomas is characterized by a high density of tumor-infiltrating lymphocytes and tumor-associated macrophages [10]. T-cells have been reported to dominate the TME across all subtypes of pituitary adenomas, while recent studies have shown that CD68 + macrophages predominate the immune infiltration in somatotropinomas [6, 10, 14]. In some studies, CD68 + macrophages and CD8 + T-lymphocytes have been linked to growth patterns of somatotropinomas and response to first-line medical therapy with first-generation somatostatin receptor ligands (SRLs) [14–16]. Specifically, higher CD68 + macrophage infiltration has been correlated with increased tumor volume, higher Ki-67%, and cavernous sinus invasion in somatotropinomas [15].

However, despite these initial efforts, the precise composition and function of the immune landscape in somatotropinomas remains to be fully elucidated, particularly concerning the expression of genes involved in TME regulation. To date, no studies have been conducted to establish a correlation between genetic alterations and immune components of the TME in somatotropinomas. Thus, this study aims to investigate the existence of genes that may regulate immune cell infiltration in the TME of patients with acromegaly.

## Materials and methods

A retrospective, longitudinal, observational, single-center study was conducted at the Pituitary Unit of the Department of Endocrinology and Metabolic Diseases of Gemelli University Hospital in Rome.

### Patients

After providing informed consent, patients with acromegaly followed up at our center were consecutively enrolled in this study if they met all the following inclusion criteria:

- Age of 18 years or older;
- Patients affected by sporadic acromegaly [3];
- Patients without a family history of pituitary adenomas and other tumors that might be part of a syndrome involving the pituitary or other endocrine glands;
- Patients with acromegaly considered clinically aggressive, such as patients diagnosed with acromegaly before 30 years old and carrying an invasive tumor, or patients not-responsive to fg-SRLs, or patients carrying multiple comorbidities related to acromegaly [5].

Patients were excluded from the study if:

- Carriers of germline mutations for the following genes: AIP, PRKAR1A, GPR101, GNAS, MEN1, CDKN1B, SDHx, MAX [17];
- Clinically diagnosed for multiple endocrine neoplasia type 1 or type 4, McCune–Albright syndrome, Carney complex or phaeochromocytoma/paraganglioma-pituitary adenoma association, familial isolated pituitary adenoma (FIPA) [17];
- Had a family history of pituitary adenomas and other tumors that might be part of a syndrome involving the pituitary or other endocrine glands, such as thyroid tumors or neuroendocrine tumors of the lungs, or gastrointestinal tract, pancreas, or thymus; pheochromocytoma or paragangliomas; neurofibromatosis; pulmonary blastomas; skin changes suggestive of Carney complex or neurofibromatosis.

### Controls

Subjects included in control group were consecutively selected if presented:

- Age of 18 years or older;
- Clinical Exome Sequencing (CES) analysis results, which was performed to search for mutations in the context of other types of tumors (i.e. breast, ovary, bone, kidney), excluding pituitary adenomas.

Subjects were excluded from the study if:

- Carriers of germline mutations for the following genes: AIP, PRKAR1A, GPR101, GNAS, MEN1, CDKN1B, SDHx, MAX, and other genes related to pituitary adenomas [17];
- Clinically diagnosed for multiple endocrine neoplasia type 1 or type 4, McCune–Albright syndrome, Carney complex or phaeochromocytoma/paraganglioma-pituitary adenoma association, familial isolated pituitary adenoma (FIPA), and other syndromes related to pituitary adenomas [17].

### Data sources and collection

Data on demographic, clinical, hormonal, molecular features, and morphology of somatotropinomas were retrospectively collected from the medical records of the patients that were included in the study (Supplementary Table 1).

For patients with acromegaly, the following informations were collected: gender, age, biochemical parameters including IGF-I values at diagnosis, tumor morphology (dimension, pattern of growth/invasion), tumor molecular characteristics, such as pituitary hormones expression, somatostatin receptor subtype 2 expression (SSTR2A), somatostatin receptor subtype 5 expression (SSTR5), cytokeratin pattern (sparsely or granular or intermediate), proliferative index percentage (Ki67%), according to the 5th WHO Classification (2022) of endocrine and neuroendocrine tumors [4].

### DNA extraction and library preparation

Germline DNA was extracted from blood samples using the MagPurix Blood DNA Extraction kit 200 (Zinexts Life Science - Taiwan). The DNA libraries were prepared using the CES V2 kit (SOPHiA GENETICS, Lausanne, Switzerland), according to the manufacturer’s protocol. This panel covers 4490 genes’ coding regions related to known inherited disease-causing mutations. The quality and quantity, as well as integrity, of DNA were estimated using the TapeStation system (Agilent Technologies, Santa Clara, CA, USA) and Qubit^®^ dsDNA HS assay kit on Qubit^®^ Fluorometer 4.0 (Invitrogen Co., Life Sciences, Carlsbad, USA), respectively. In each Next Generation Sequencing (NGS) run, an equimolar pool of all library samples was prepared and loaded to a final concentration of about 1.8 pM and 3% Phix. The sequencing was performed using NextSeq500/550 Mid output kit (300 cycles) on Illumina NextSeq550 Dx (Illumina, San Diego, CA, USA) platform.

### Bioinformatics analysis

**Fig. 1:**
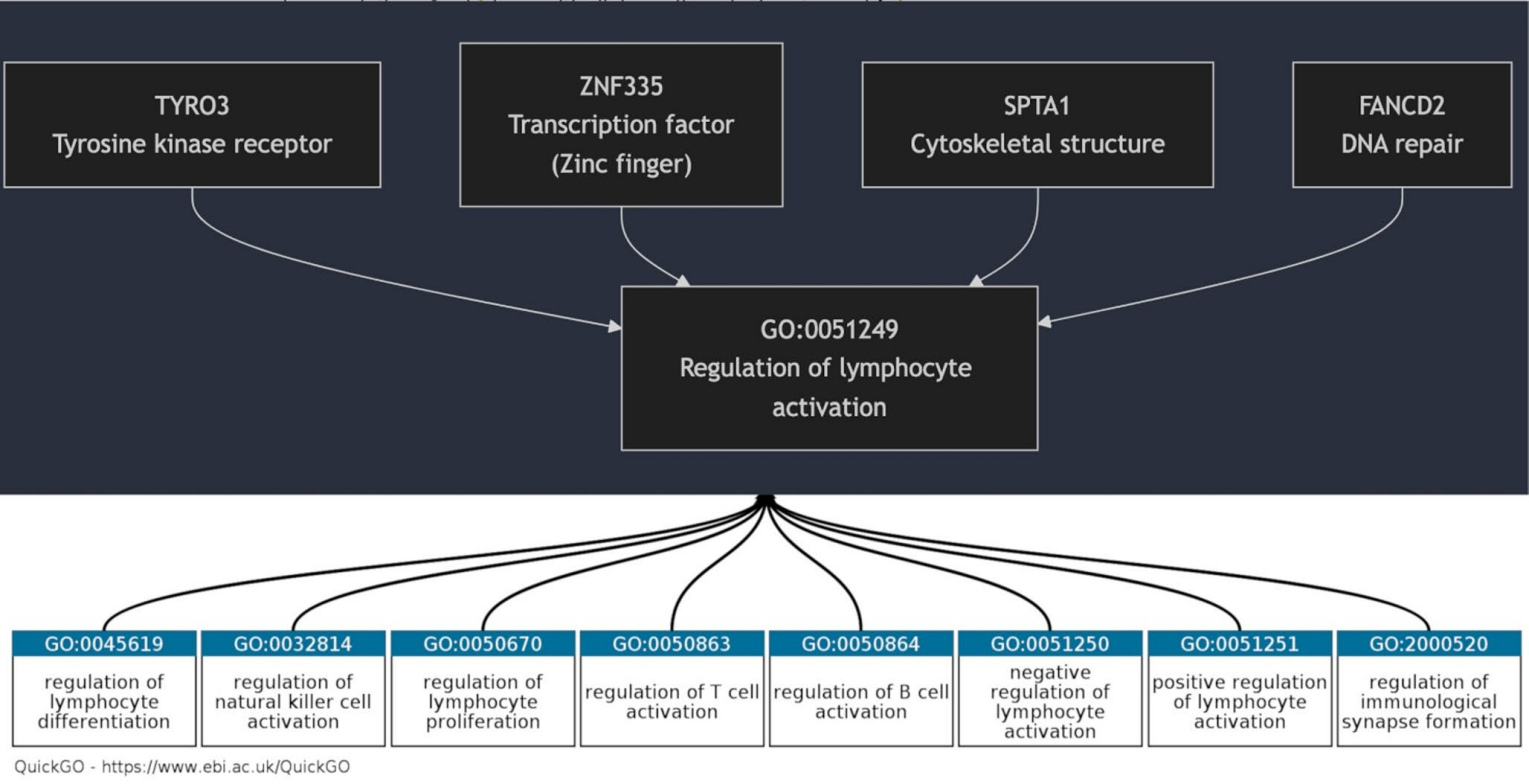
Overview of the functional associations between four candidate genes and the biological processes defined by GO:0051249 (“Regulation of lymphocyte activation”). The upper panel displays the network of genes (TYRO3, ZNF335, SPTA1, FANCD2) identified through STRING analysis, while the lower panel illustrates the corresponding GO terms related to lymphocyte activation. This integrated representation highlights the potential regulatory role of these genes in modulating immune cell activation

At the end of each run, Fastq files were collected and uploaded on the SoPHIA DDM software for mapping (bam files) and variant calling (vcf files) steps. At this stage raw sequence data underwent quality control (QC) for filtering and low-quality reads removal. Additionally, adapter trimming was performed to ensure the integrity of the data. The SOPHiA DDM™ Platform was used for detecting, annotating and pre-classifying multiple types of genomic alterations with a dedicated pipeline. Plots, tables and graphs were all generated using RStudio software embedded with R v.4.3.3. As reported in Table S1 and Figure 1, after checking data integrity (step 1) and selecting variants with a depth of coverage of at least 30x (step 2), all variants classified as benign and/or likely benign by ClinVar (step 3) were discarded (30.8% of all raw variants). The remaining 230,172 variants were further filtered out if the allele frequency was lower than 30% and filters of SOPHiA DDM pipeline were applied (step 4). Intronic, synonymous as well as intergenic (steps 5–7) variants were also removed. Then, population filters (MAF < 0.05) from 1000 Genomes and ExAC were applied in steps 8–9. Finally, ENSEMBL Impact classification was applied to the remaining variants (step 10). High, moderate and modifier variants were selected, thus retaining 0.8% (18257 records) of the starting dataset (100%, 2324119 variants).

SOPHiA DDM™ Platform incorporates a register of the allelic variants called in all patients/subjects who are consecutively subjected to genetic analysis. As NGS is developed within the scope of various diagnostic projects, the database of SOPHIA allowed us to match genomic data of our patients against overall individual screened, underlying the differences between our acromegalic cohort and the other individuals screened for other diseases/the latter as aggregate data.

### Bioinformatics tools and databases

#### Pipeline

In step 10 of our ad hoc bioinformatics pipeline we included from Ensembl GRCh37 Release 112 (May 2024) the Ensembl Variant Effect Predictor (VEP) module to retain variants classified as high, moderate or modifier (https://grch37.ensembl.org/index.html).

#### Tertiary analysis

Meanwhile, downstream analyses were performed using a set of dedicated tools and databases. In compliance with American College of Medical Genetics and Genomics (ACMG) guidelines [18]: (a) we collect meta scores from VARSOME to evaluate variants’ pathogenicity based on the combined evidence of multiple in-silico predictors (https://varsome.com/); (b) Franklin (https://www.hgmd.cf.ac.uk/ac/index.php) and ClinVar (https://www.ncbi.nlm.nih.gov/clinvar/) databases were used to classify variants into five categories (Benign, Likely benign, Variant of Uncertain Significance, Likely Pathogenic and Pathogenic) using ACMG pathogenicity criteria.

#### Gene-disease and pathway analyses

Genes associated with pituitary adenomas were identified using GeneCards, DisGeNET, and Online Mendelian Inheritance in Man (OMIM) databases. The intersection of genes between analyzed groups was determined using Venny 2.1. The Metascape database was employed to perform pathway enrichment analyses of Gene Ontology (GO) biological process [19–23] and Kyoto Encyclopedia of Genes and Genomes (KEGG) Pathway for the targets [24].

A generalized linear model (GLM) regression analysis was used to select those genes with remarkable divergence in the number of subjects (both patients and controls) across the overall mutational loads per gene. We defined and ranked a list of top 40 genes. These were selected and further investigated to address potential associations with pathways of interest, according to the tumor characteristics. Enrichment analysis was performed considering available clinical features associated with acromegaly.

#### Histopathological assessment

Histopathological assessment was conducted according to our clinical practice on somatotropinoma samples of all the patients enrolled in the study. All specimens were examined for pituitary hormones, Ki-67, p53, pituitary-specific transcription factor (Pit-1), and TME components. The Ki-67 index was expressed as percentage of positive nuclei in hot spot. The number of CD8 + lymphocytes and CD68 + macrophages was expressed as the average of positive cells in four high-power fields (HPF, 0.8 mm2). Fields were randomly selected within somatotroph tumor tissue. Fields were not analyzed if sited close to vessels or in areas of doubt, such as the interface between the tumor and the normal pituitary gland. Appropriate positive control slides with immunohistochemistry for CD45 cells were included for each staining, while one section was processed with the omission of the primary antibody as a negative control. The positive cells of four sequential fields were counted. Cells were considered positive only if the cellular nucleus was identified.

### Statistical analysis

Descriptive statistical analyses included median and interquartile ranges (IQR) for continuous variables. The Mann–Whitney and Spearman correlations were used for continuous variables. Nonparametric tests were employed because the data were not normally distributed. For qualitative variables, absolute and relative frequencies were reported and the Fisher’s exact test was applied. Statistical significance was assumed for *p* < 0.05. The data were analyzed using SPSS Software, version 22.

## Results

CES analysis was performed on 46 patients diagnosed with acromegaly (mean age ± SD = 55 ± 12.3 years; 31 females), and 39 patients as controls (mean age ± SD = 52 ± 15.7 years; 28 females). 9864 unique variants were identified in 3108 genes, of which 5759 were found in patients with acromegaly. The most frequently mutated genes were: SLC646, FDFT1, KCNJ12, RNF212, TTN, OVCH2, ATXN3, COL18A1, SFTPA1, TRIOBP, DLGAP3, MUC5B, SYN2, OBSCN, TNXB, SHANK3, ZFHX3, TRAK1, KCNN3, RP1L1, ZNF469, ZAN, TG, РКD1, APOB, TYRO3, SYNE2, MSH3, SYNE1, PLEC, FANCD2, DYSF, DMXL1, CELSR1, DGCR2, ALMS1, SIGLEC14, ABCA13, SPTA1, PTGIS.

Enrichment analysis was performed on the entire set of genes included in our panel. Selecting the top 40 above-mentioned genes, we investigated the most relevant pathway with a FDR value below < 0.05 according to STRING analysis default protocol. Interestingly, 144 genes of pathway GO:0051249 (regulation of lymphocyte activation) were available in our panel, indeed 4 of these were found with an overall higher mutational load compared to other genes belonging to the same pathway (Fig. 1).

**Fig. 2:**
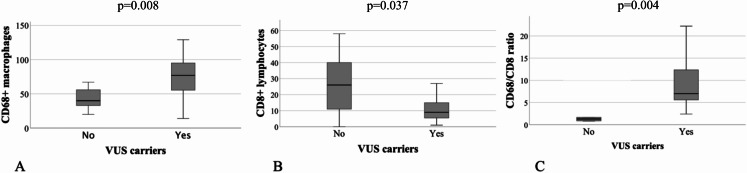
**A**: Box-plot representing the number of CD68 + macrophages infiltering somatotropinomas in patients carriers of VUS of “regulation of lymphocyte activation” genes. **B**: Box-plot representing the number of CD8 + T-lymphocytes infiltering somatotropinomas in patients carriers of VUS of “regulation of lymphocyte activation” genes. **C**: Box-plot representing the CD68+/CD8 + ratio in somatotropinomas in patients carriers of VUS of “regulation of lymphocyte activation” genes

**Fig. 3:**
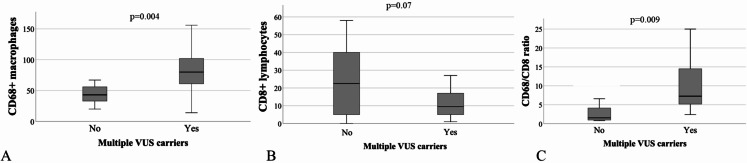
**A**: Box-plot representing the number of CD68 + macrophages infiltering somatotropinomas in patients carriers of multiple VUS of “regulation of lymphocyte activation” genes. **B**: Box-plot representing the number of CD8 + T-lymphocytes infiltering somatotropinomas in patients carriers of multiple VUS of “regulation of lymphocyte activation” genes. **C**: Box-plot representing the CD68+/CD8 + ratio in somatotropinomas in patients carriers of multiple VUS of “regulation of lymphocyte activation” genes

33 patients out of the 46 that were included in the study (72%) showed the presence of at least one variant of unknown significance (VUS) in at least one of the following genes: FANCD2, SPTA1, TYRO3, and ZNF335 (Table 1). The enrichment pathway analysis of these genes was performed on Gene Ontology and showed that FANCD2, SPTA1, TYRO3, and ZNF335 genes were all included in the same genetic pathway called “regulation of lymphocyte activation” (GO:0051249). To investigate the relationship between genetic background and immune TME, we investigated the immune cell infiltration in 26 patients, with available histological samples of somatotropinomas. In this cohort 22 were females (84.6%), median age at acromegaly diagnosis was 48 years (IQR: 19.7). All patients carried GH-secreting macroadenomas (100%), that were invasive in 11 cases (42.3%). The proliferative index was higher than 3% in 9 patients (34.6%). 9 patients were cured through surgery (34.6%), 6 patients were controlled during treatment with first generation somatostatin receptor ligands (SRLs) (23.1%), 11 patients were controlled during treatment with GH receptor antagonist (Pegvisomant) or second generation SRLs (Pasireotide Lar). As shown in Table 2, the number of CD68 + macrophages was significantly higher in samples of patients with germline variants of genes of “regulation of lymphocyte activation” pathway (48/HPFs; IQR:30), in comparison to acromegaly patients without any genetic alterations (40/HPFs; IQR: 28; p-value = 0.008), while the CD8 + T-lymphocytes number was reduced in mutated patients (8.5/HPFs; IQR: 5), compared to wild-type patients (37/HPFs; IQR: 28; P-value = 0.037). Also, the CD68+/CD8 + ratio was significantly higher in samples of mutated patients (6.4; IQR: 8.5; P-value = 0.004), whereas the analysis on CD4 + T lymphocytes yielded no statistically significant results, as reported in Fig. 2. Finally, patients with multiple VUS presented a significantly higher number of CD68 + macrophages (single VUS: median 40; IQR: 19; multiple VUS: Median 80; IQR: 46; p-value = 0.004) and the CD68+/CD8 + ratio resulted significantly higher in patients with multiple VUS (Single VUS median: 7.27; IQR: 11.4. multiple VUS; median 1.5; IQR: 5.7. p-value = 0.009). Instead, the number of CD8 + lymphocytes did not differ among patients carrying a single or multiple VUS (*p* = 0.07), as reported in Fig. 3. Instead, gender, age, IGF-I and GH levels at diagnosis, tumor dimension, invasive growth, cytokeratin pattern, and overall disease outcome did not differ among patients carrying a single VUS compared to patients carrying multiple VUS. Interestingly, in this cohort of patients, the number of tumor-infiltrating CD8+, CD68+, and the CD68+/CD8 + ratio did not differ in invasive and non-invasive tumors and with the overall outcome of the disease.

**Table 1:** List of variants (VUS) in FANCD2, SPTA1, TYRO3, and ZNF335 genes included in the “regulation of lymphocyte activation” pathway (GO:0051249) found in our cohort of patients with acromegaly

Genes	Variants cDNA (RefSeq)	Protein consequence
FANCD2	NM_001018115:c.230 A > C	p.Lys77Thr
FANCD2	NM_001018115:c.2965 C > G	p.Pro989Ala
SPTA1	NM_003126:c.4265 A > G	p.Asp1422Gly
SPTA1	NM_003126:c.1250 A > G	p.His417Arg
SPTA1	NM_003126:c.122G > A|	p.Arg41Gln
TYRO3	NM_001330264:c.2147 + 3_2149del	
TYRO3	NM_006293|c.86_109del	p.Ala29_Pro36del
TYRO3	NM_001330264:c.-10_0del	
TYRO3	NM_001330264:c.173 + 1_174-1del	
TYRO3	NM_001330264:c.1740 + 1_1741-1del	
TYRO3	NM_001330264:c.1850 + 2_1851del	
TYRO3	NM_001330264:c.2010 + 1_2011-1del	
TYRO3	NM_001330264:c.1247 + 2_1248del	
TYRO3	NM_001330264:c.1348 + 1_1349-1del	
TYRO3	NM_001330264:c.1618 + 2_1619del	
TYRO3	NM_001330264:c.1117 + 2_1118del	
TYRO3	NM_001330264:c.274 + 4_277del	
TYRO3	NM_001330264:c.826 + 1_827-1del	
TYRO3	NM_001330264:c.972 + 2_973del	
TYRO3	NM_001330264:c.1111 C > T	p.Arg371Cys
TYRO3	NM_001330264:c.1349-1G > T	
TYRO3	NM_001330264:c.1444 + 2_1445del	
ZNF335	NM_022095:c.1855 C > T	p.Arg619Cys
ZNF335	NM_022095:c.1513 C > T	p.Arg505Cys

**Table 2:** Demographic and clinical characteristics of acromegaly patients; morphology, molecular, and immune cells infiltration characteristics according to the “regulation of lymphocyte activation” pathway (GO:0051249)

	Wild-type patients	VUS carrier patients	*P*-value
Number of patients (%)	7 (26.9%)	19 (73.1%)	n.a
Gender			
Males n, (%) Females n, (%)	1 (25%) 6 (27.3%)	3 (75%) 16 (72.2%)	0.713
Age years, median (IQR)	50 (11.2)	50.5 (11.2)	0.574
GH ng/mL median (IQR)	11.3 (17.7)	6.1 (11.6)	0.733
IGF-I x ULN median (IQR)	3 (2.6)	2.2 (1.3)	0.189
Tumor dimension			
Macroadenoma n, (%)	7 (28%)	18 (72%)	n.a
Invasive growth			
No, n (%) Yes n, (%)	3 (20%) 4 (36.4%)	12 (80%) 7 (63.6%)	0.313
Proliferative Index			
MIB1 *≤* 3% n, (%) MIB1 > 3% n, (%)	5 (29.4%) 2 (22.2%)	12 (70.6%) 7 (77.8%)	0.483
Outcome			
Cured n, (%) Responsive fg-SRLs n, (%) Resistance to fg.SRLs n, (%)	2 (22.2%) 0 (0%) 5 (45.5%)	7 (77.8%) 6 (100%) 6 (54.5%)	0.795
CD8+/HPFs median (IQR)	37 (28)	8.5 (5)	0.037
CD4+/HPFs median (IQR)	7 (11)	3 (5)	0.796
CD68+/HPFs median (IQR)	40 (28)	48 (30)	0.008
CD68+/CD8 + ratio median (IQR)	1 (0.8)	6.4 (8.5)	0.004
CD8+/CD4 + ratio median (IQR)	8.2 (16)	2.5 (4.1)	0.239

## Discussion

The TME is a milieu derived from the interaction between tumour cells and the host immune system, with the ability to modulate tumor proliferation, invasiveness, and aggressiveness [7, 25, 26]. The immune cells that predominantly characterize the TME of somatotropinomas are TAMs and TILs [10, 27–30]. According to recent studies, the total number of CD8 + T lymphocytes tends to be lower in invasive, large tumors with cavernous sinus invasion and in those resistant to first-line medical therapy, underpinning a more favorable prognosis [14, 16]. Moreover, previous data from our centre revealed that a higher number of CD68 + macrophages positively correlates with tumor size, invasiveness and cancer cells proliferation, shaping macrophages as possible markers of tumor cell proliferation and, consequently, of worsened prognosis [15]. Our study focused exclusively on sporadic GH-secreting PAs, revealing a correlation between the infiltration of CD68 + macrophages and specific genetic modifications. An intriguing prior study on familial pituitary adenomas has demonstrated the presence of CD68 + macrophage infiltration associated with AIP genetic mutations, suggesting that the interplay between genetic factors and the infiltration of inflammatory cells within adenoma tissue could exhibit a broader spectrum of implications, either in hereditary or sporadic context [31]. This study revealed, for the first time, a possible correlation between the specific genetic pathway “regulation of lymphocyte activation” (GO:0051249) and immune cell infiltration in somatotropinomas. According to Gene Ontology available data, this pathway is involved in the modulation of the activation of lymphocytes, a fundamental regulatory process in maintaining a balance between an effective immune response and the prevention of autoimmune reactions. Among the genes included in this pathway, recent studies have suggested that FANCD2, SPTA1, TYRO3, and ZNF335 genes are emerging as key players in immune response and regulating tumorigenesis [32–38], even if their role has not been studied in TME of somatotropinomas. The FANCD2 (Fanconi Anemia Complementation Group D2) gene is involved in the regulation of ferroptosis and DNA repair, through the activation of cell cycle checkpoints following DNA damage [39]. Regulation of FANCD2 by the mTOR pathway, a molecular pathway known to contribute to cancer cell resistance to DNA damage also in pituitary adenoma, increases tumor cell survival. Overexpression of FANCD2 has been identified as a potential mechanism by which tumor cells may evade therapeutic intervention [33]. FANCD2 and FANCI complex is involved in the repair of DNA to maintain cell survival. High expression of FANCI has been associated with a poor prognosis in cancer, as proved in lung adenocarcinoma [32], as it promotes tumor growth by suppressing M1 macrophages, which play an anti-tumor role in the immune response [40]. Consequently, these findings emphasize the ability of FANCD2 to modulate macrophage differentiation in different tumor types, thereby proposing the hypothesis that a similar phenomenon may occur in somatotropinomas [41]. SPTA1 (Spectrin Alpha, Erythrocytic 1) is a cytoskeletal protein that connects the plasma membrane to the actin of the cytoskeleton, playing a central role in preserving cellular shape and structural integrity [42]. Furthermore, it is involved in the organization of transmembrane proteins and cellular response to external signals, regulating cell migration and proliferation [42]. SPTA1 may potentially influence the TME by promoting tumor cell invasiveness and modulating the interaction between tumor cells and surrounding stromal cells and consequently enhance tumor dissemination of tumors [43–45]. TYRO3, also known as Tyrosine-Protein Kinase Receptor TYRO3, is a member of the TAM (TYRO3, Axl, and Mertk) receptor tyrosine kinase family [34]. In the present study, TYRO3 was identified as the most mutated gene in the cohort under investigation. The function of TAM family is to regulate immune responses, cell survival, and tissue repair. TYRO3 activation, through the process of phosphorylation, leads to the activation of downstream signaling pathways such as PI3K/AKT and MAPK, which are known to promote cell proliferation and survival in PAs [6, 46–49]. TYRO3 plays a substantial influence on immune responses, notably by modulating the polarization of macrophages towards the pro-tumorigenic M2 phenotype [35]. This shift can support tumor growth and immune evasion, similar to FANCD2. Furthermore, TYRO3 has been demonstrated to inhibit ferroptosis, as well as FANCD2, thereby enhancing tumour cell survival. The ZNF335 gene, encoding the transcription factor ZNF335 (Zinc Finger Protein 335), is critical for the proper regulation of gene expression through the interaction with histone methyltransferase complexes. It plays a crucial role in the processes of neurogenesis and the regulation of neural progenitor cell proliferation [50]. In the context of tumors, it was proposed that the zinc finger protein supports the development of a pro-tumorigenic TME, similar to TYRO3, by promoting the polarization of macrophages toward the M2 phenotype and further enhancing tumor progression [51].

**Fig. 4:**
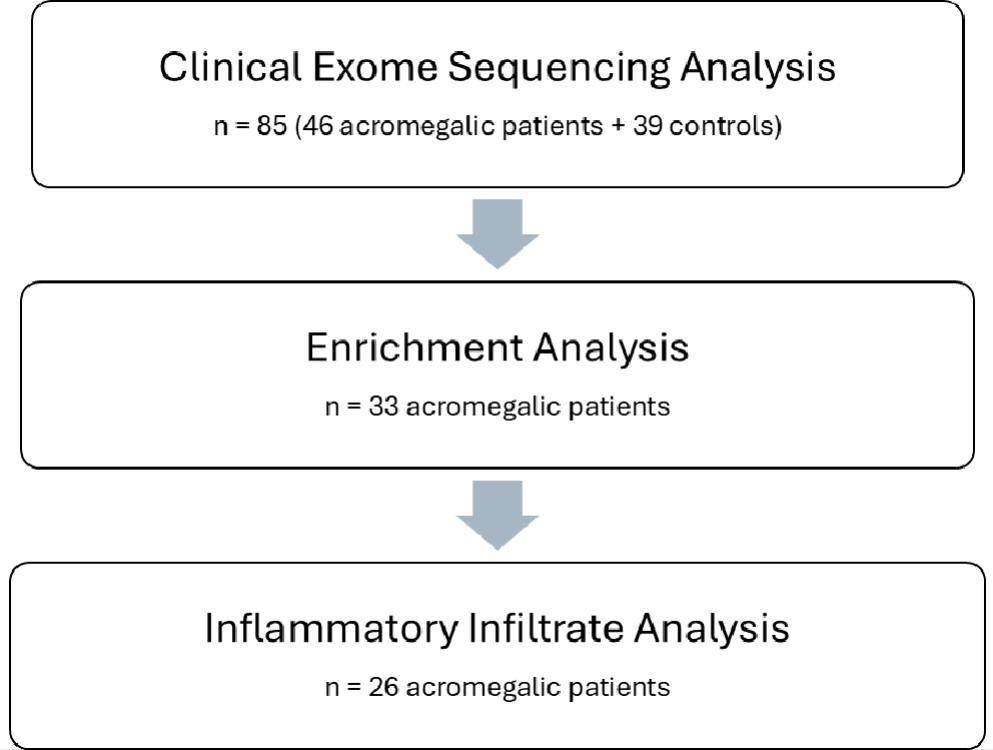
Panel representing the selection process of patients with acromegaly and controls

In summary, this study revealed an intricate interplay between genes of the “regulation of lymphocyte activation” pathway and immune cells infiltration in somatotropinomas, underlying the complex dynamics of the TME in acromegaly. As in other types of tumors these genes may contribute to the pro-tumorigenic TME, by influencing tumor cell survival, immune cell activation, and macrophage polarization. A more comprehensive understanding of the roles of these specific genes within the context of the TME could potentially offer significant insights into the molecular basis of somatotropinomas heterogeneity. The retrospective nature of the study, the small sample size, the absence of functional characterization of the variants and the absence of family segregation studies are acknowledged as limitations of this study. Moreover, according to the different design and aim of this study (Fig. 4), the different classification of disease outcomes and the limited number of patients, the prognostic role of tumor infiltrating CD8 + and CD68 + immune cells was limited in this report with respect to previous ones that we have conducted [14, 15]. To date, available studies defining the immune cell infiltration in somatotropinomas have been performed on small sample sizes, given the rarity of acromegaly. The retrospective design of the study precluded the investigation of further TME elements. Excluding patients with a family or hereditary history of somatotropinomas, or associated syndromes, reduced the probability of finding pathogenic variants; however, this also led to a more homogeneous sample of patients with acromegaly. Additionally, as the search for genetic variants was confined to genes that have previously been reported to be associated with germline, mosaic or somatic pituitary adenomas, this may have exerted a constraining effect on the identification of novel genetic associations. Future studies will be required to confirm our findings and address these limitations. In conclusion, this study suggests that genes included in the genetic pathway “regulation of lymphocyte activation” may influence immune cell infiltration in acromegaly. These findings offer new insights into the genetic basis of the TME in somatotropinomas and the genes presented may be of interest for a more in-depth study of gene regulation of the TME in acromegaly.

## Electronic supplementary material

Below is the link to the electronic supplementary material.

Supplementary Material 1

## Data Availability

No datasets were generated or analysed during the current study.
