# Supplementary material for: Genes of the “regulation of lymphocyte activation” pathway may influence immune cells infiltration in growth hormone secreting pituitary tumors
Source: Pituitary. 2025 May 26;28(3):63. doi: 10.1007/s11102-025-01537-w (PMC12104112; doi:10.1007/s11102-025-01537-w)
Supplement: Supplementary file 1 — Supplementary Material 1 [file 11102_2025_1537_MOESM1_ESM.docx]

**Supplementary Table 1. General and clinical characteristics of patients with acromegaly (n=46)**

|  |  |  |  | |
| --- | --- | --- | --- | --- |
| **Gender** |  |  | *(n=46)* | |
|  |  | *Female* | 31 (67%) | |
|  |  | *Male* | 15 (33%) | |
|  |  |  |  | |
| **Age** |  |  | *Mean age ± SD* | |
|  |  | *Years* | 55 ± 12.4 | |
|  |  |  |  | |
| **Tumor characteristics** | |  | *(n=46)* | |
|  | ***Age at diagnosis*** | *Late (>30 y.o.)* | 40 (87.0%) | |
|  |  | *Early (<30 y.o)* | 6 (13.0%) | |
|  | ***Tumor dimension*** | *<1 cm* | 11 (23.9%) | |
|  |  | *>1 cm* | 35 (76.1%) | |
|  | ***Cavernous sinus invasiveness*** | *No* | 28 (61.0%) | |
|  |  | *Yes* | 18 (39.1%) | |
|  | ***Ki67%*** | *< 3%* | 25 (54.4%) | |
|  |  | *> 3%* | 9 (19.6%) | |
|  |  | *n.a.* | 12 (26.1%) | |
|  | ***Mixed**** | *GH-secreting tumor* | 21/43 (48.8%) | |
|  |  | *Mixed PitNET tumor* | 22/43 (51.2%) | |
|  |  |  |  | |
| **Clinical data** |  |  | *No.* | |
|  | ***IGF-I level (ng/mL)*** | *Median (Q1-Q3)* | 788 (422– 949) | |
|  |  |  |  |  |

*Data available only for the 43 patients who underwent surgery.
Abbreviations: *n.a.*, data not available; *No*. number of related subjects; *SD*, standard deviation
